# Expression pattern of the thrombopoietin receptor (Mpl) in the murine central nervous system

**DOI:** 10.1186/1471-213X-10-77

**Published:** 2010-07-28

**Authors:** Anna Ivanova, Jens Wuerfel, Juan Zhang, Olaf Hoffmann, Matthias Ballmaier, Christof Dame

**Affiliations:** 1 Department of Neonatology, Charité - Universitätsmedizin, Germany; 2 Institute of Neuroradiology, Universität Lübeck, Germany; 3 Department of Experimental Neurology, Charité - Universitätsmedizin Berlin, Germany; 4 Department of Neurology, St. Josef Krankenhaus, Potsdam, Germany; 5 Department of Paediatric Haematology and Oncology, Medizinische Hochschule Hannover, Germany

## Abstract

**Background:**

Thrombopoietin (Thpo) and its receptor (Mpl), which regulate megakaryopoiesis, are expressed in the central nervous system (CNS), where Thpo is thought to exert pro-apoptotic effects on newly generated neurons. Mpl expression has been analysed in brain tissue on transcript level and in cultured primary rat neurons and astrocytes on protein level. Herein, we analysed Mpl expression in the developing and adult murine CNS by immunohistochemistry and investigated the brain of mice with homozygous *Mpl *deficiency (*Mpl*^-/-^) by MRI.

**Results:**

Mpl was not detectable at developmental stages E12 to E15 in any resident cells of the CNS. From E18 onwards, robust Mpl expression was found in various brain areas, including cerebral cortex, olfactory bulb, thalamus, hypothalamus, medulla, pons, and the grey matter of spinal cord. However, major developmental changes became obvious: In the subventricular zone of the cerebral cortex Mpl expression occurred only during late gestation, while in the hippocampus Mpl expression was detectable for first time at stage P4. In the white matter of the cerebellum Mpl expression was restricted to the perinatal period. In the adult cerebellum, Mpl expression switched to Purkinje cell. The majority of other Mpl-positive cells were NeuN-positive neurons. None of the cells could be double-labelled with astrocyte marker GFAP. *Mpl*^-/- ^mice showed no gross abnormalities of the brain.

**Conclusions:**

Our data locate Mpl expression to neurons at different subdivisions of the spinal cord, rhombencephalon, midbrain and prosencephalon. Besides neuronal cells Mpl protein is also expressed in Purkinje cells of the adult cerebellum.

## Background

Thrombopoietin (Thpo) and its receptor (Mpl), which primarily regulate megakaryopoiesis, are also expressed in the central nervous system (CNS). Although a 39 amino-acid fragment of the N-terminal domain of Thpo shares significant homology with erythropoietin (Epo), which acts as neuroprotective substance [1], and with various neurotrophins [2], current data show pro-apoptotic effects of recombinant Thpo on newly generated neuronal cells in models of brain hypoxia/ischemia [3].

Expression of Thpo transcripts and/or protein has been described in the developing and adult brain, including the cerebral cortex, hippocampus, brain stem, and cerebellum [3-7]. Thpo mRNA and protein have been detected in neurons, astrocytes and microglia derived from the cerebral cortex and the hippocampi, as well as in Purkinje cells and in granule cells of the cerebellum [3,6]. Thpo seems to be involved in the inflammatory response of the brain, because some patients with meningitis demonstrated elevated Thpo levels in the cerebrospinal fluid [8-10]. Thpo is most likely produced locally, since its high molecular weight (about 70-80 kDa) makes its crossing over the intact blood-brain barrier rather unlikely [11]. Under experimental conditions of hypoxia, *Thpo *mRNA expression is down-regulated [3,6,8].

Thpo is thought to mediate its effects by binding to its receptor Mpl. However, data on the expression and regulation of Mpl in the CNS are very limited. So far, expression of *Mpl *mRNA has been described in the CNS of mice, rats and humans [3,4]. Region-specific analysis revealed *Mpl *transcripts in the adult rat cortex and hippocampus. In primary rat neurons and astrocytes, Mpl expression has been confirmed by RT-PCR and immunostaining. Under hypoxia *Mpl *mRNA levels increased in astrocytes, but decreased in hippocampal neurons, suggesting a differential regulation under certain conditions [3]. Additionally, we recently showed that Mpl is expressed on transcript and protein levels in cultured brain-derived endothelial cells of adult mice [12].

Neither malformation nor functional deficits of the CNS have yet been reported in transgenic mice with homozygous deletion of the *Thpo *(*Thpo*^-/-^) or *Mpl *(*Mpl*^-/-^) gene [13-15]. However, cerebral and cerebellar hypoplasia have been described in some patients with congenital amegakaryocytic thrombocytopenia (CAMT) [16,17], which is caused by mutations in the ligand-binding domain of the MPL. In the thrombocytopenia and absent radii (TAR) syndrome, for which a defect in Mpl signalling has been shown [18], cerebellar dysgenesis either with/without agenesis of the corpus callosum and delayed myelinisation have been reported [19-21].

Herein, we analysed the Mpl expression pattern in the developing and adult murine CNS by immunohistochemistry in order to highlight cells sensitive to Thpo and to identify the time frame for the highest activity of the Thpo/Mpl system.

## Results

### Specificity of the Mpl antibody

**Figure 1:**
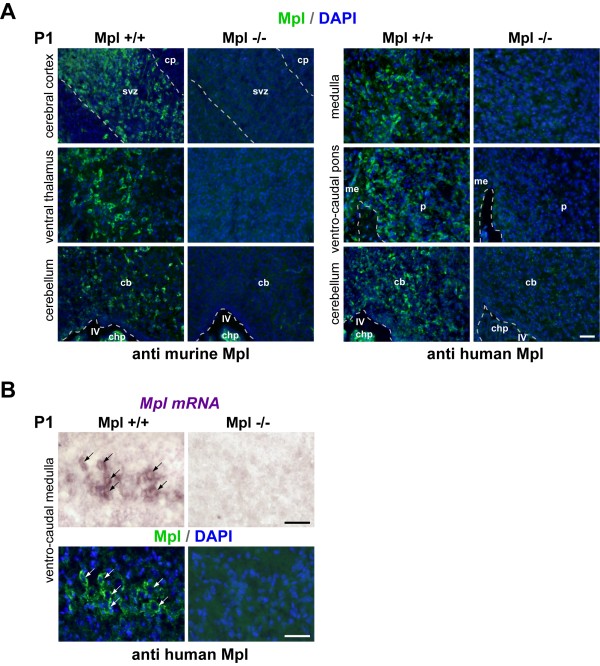
**Validation of the Mpl antibody**. (A) Comparison of two antibodies recognizing Mpl on cryosections from developing *Mpl*^-/- ^and wild-type mice one day after birth. Left panel: rat monoclonal anti-mouse Mpl (Kirin Brewery), right panel: murine monoclonal anti-human Mpl (R&D Systems). Specific labelling was observed in the brain tissue from wild-type mice (left columns), but was absent in *Mpl*^-/- ^mice (right columns). (B) Combined *in situ *hybridization (top row) and immunolabelling (bottom row) on the same sections show that in wild-type mice (left column) the anti-human Mpl antibody (R&D Systems) recognizes the same cells which are labelled by the riboprobe directed against mouse *Mpl *mRNA. Arrows indicate double-labelled cells. Specific labelling is absent in the corresponding region of the *Mpl*^-/- ^mouse (right column). cb - cerebellum; chp - choroid plexus; cp - cortical plate; me - medulla; p - pons; svz - subventricular zone; IV - forth ventricle. Scale bars: 50 μm in all panels.

To test the different anti-Mpl antibodies for specificity in the brain, we compared the recognition pattern between wild-type and *Mpl*^-/- ^mice. A monoclonal antibody directed against the murine Mpl (clone AMM2; Kirin Brewery) and a commercially available antibody directed against human Mpl (anti-human TpoR, clone 167639, R&D Systems) both displayed specific labelling of brain parenchymal cells of neonatal wild-type mice, but not in *Mpl*^-/- ^pups (Figure 1A). Labelling of the choroid plexus may be unspecific (Figure 1A) as often the case in this structure. Of note, consecutive sections were also stained without using the primary antibody. Additionally, we confirmed the specificity of the anti-human Mpl antibody by comparing *in situ *hybridization and immunohistochemistry in the same section of the neonatal wild-type or *Mpl*^-/- ^brain (Figure 1B). Of note, various clones of this anti-human antibody differed in the quality of staining, and the best results were obtained using the 167639 clone. Because of the commercial availability of this antibody, we used it for the rest of the analyses. The combined testing has been quoted as a golden standard for validation of antibodies to be used in immunohistochemistry [22].

### Expression pattern of Mpl in the developing brain

**Figure 2:**
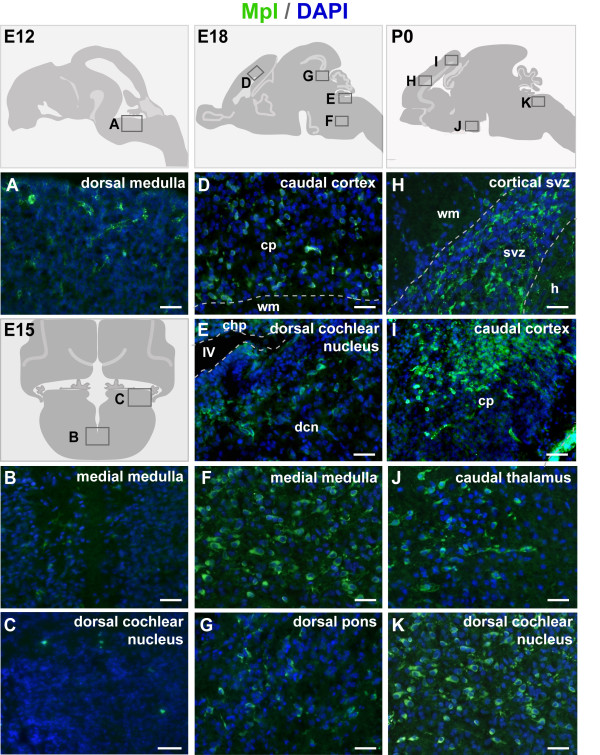
**Detection of Mpl-positive cells in the embryonic central nervous system**. Schematic drawings show the positions of the micrographs. During early embryonic development both brain and spinal cord are devoid of Mpl labelling. Left column shows representative examples at E12 (A) and E15 (B, C). The green labelling on the blood vessels is unspecific as it also labels negative control sections incubated without the primary antibody. By embryonic day 18 (E18) numerous Mpl-positive cells appear in the inner cortical plate, subventricular zone of the fourth ventricle and parenchymal regions of rhombencephalon (D-G). At P0 the number of Mpl positive cells reaches its peak, spreading throughout many brain regions (I, J, K), including the subventricular zone of the lateral ventricles (H). Dorsal is up, anterior to the left. chp - choroid plexus; cp - cortical plate; dcn - dorsal cochlear nucleus; h - hippocampus; svz - subventriclar zone; wm - white matter; IV - forth ventricle. Scale bar 50 μm.

Our analysis revealed no specific Mpl labelling in any resident cells of the CNS at developmental stages E12 and E15 (Figure 2A-C). Signals detected in the dorsal medulla at E12 may be an artefact (Figure 2A), since these structures were larger in size than normal neuroblasts at E12 and had ragged shape; they were also detected on consecutive sections incubated with secondary antibody alone.

From E18 onwards Mpl protein expression was detectable in the prosencephalon, midbrain, and rhombencephalon as well as in the spinal cord (Table 1). In the telencephalon, Mpl-positive cells were detected in the cerebral cortex, in the subventricular zone of the IV^th ^ventricle and in the inner layers of developing olfactory bulb, but not in the hippocampus and basal ganglia. Widespread Mpl expression was found in the diencephalon (thalamus and hypothalamus) and mesencephalon (superior and inferior colliculus); other areas included the pons (metencephalon), medulla (myelencephalon), and the grey matter of the cervical spinal cord (Figure 2 D-G, Table 1). There was no labelling within the lateral recesses of the secondary rhombic lip that generates the external granule layer of the cerebellum or granular cells of the vestibulo-cochlear anlage (*data not shown*).

**Table 1:** Expression pattern of Mpl protein in the developing and adult mouse brain.

Subdivisions		E18.5	P0	P4	P7	Adult
**Spinal cord**						

Cervical	Grey matter	++	++	++	+/-	++
	
	White matter	-	-	-	-	-

**Rhombencephalon**						

Myelencephalon	Roof plate, cochlear complex	+	++	+	-	++
	
	Medulla, r2-r8	+	++	+	+/-	++

Metencephalon	Pons, r1	+	++	+	+	++
	
	Cerebellar white matter	-	++	+	+	-
	
	Cerebellar cortex + external granule layer	-	-	-	-	-
	
	Purkinje cells of cerebellum	-	-	-	-	+

**Midbrain**						

Mesencephalon	Prepontine hindbrain	-	+	+	+	+
	
	Tegmentum	+	++	-	+	++
	
	Inferior colliculus	+	++	+	+/-	++
	
	Superior colliculus	++	++	+	+/-	++

**Prosencephalon**						

Diencephalon	Prethalamus	++	++	+	+/-	+
	
	Thalamus	++	++	++	+	++
	
	Pretectum	+	++	+	+	+
	
	Preoptic area	++	++	+	-	++
	
	Hypothalamus	+	++	+	+	+

Telencephalon	Basal ganglia	-	+	+	+/-	+
	
	Hippocampal formation	-	-	+/-	++	+
	
	Cerebral cortex, subventricular zone	-/+	++	-	-	-
	
	Cerebral cortex	+	+	+	+	+
	
	Olfactory bulb	+	+	-	-	+

The rough estimates of the staining intensity are presented in different brain regions between E18.5, one day before birth, early postnatal stages as well as in the adult brain.

Even considering that the staining intensity does not necessarily represent the strongest protein expression, it appeared that Mpl expression peaked in the early neonatal period. At birth the number of Mpl-positive cells increased as observed through many sections of different stages in parallel. In the telencephalon, the newly developing cortical subventricular zone contained numerous Mpl-positive cells with tangentially oriented processes, but the developing white matter was devoid of any labelling (Figure 2H). In addition, the caudal cortex contained a densely packed positive cell cluster (Figure 2I). Other clusters of Mpl-positive cells were located in the diencephalon (caudal thalamus; Figure 2J) and in the mes- and metencephalon (pons and medulla; *data not shown*). The cerebellar white matter became Mpl-positive, but the lateral recessus of the IV^th ^ventricle and granule cell streams remained Mpl-negative (*data not shown*). Furthermore, deep regions of the vestibulo-cochlear nuclei also contained Mpl expressing cells (Figure 2K).

**Figure 3:**
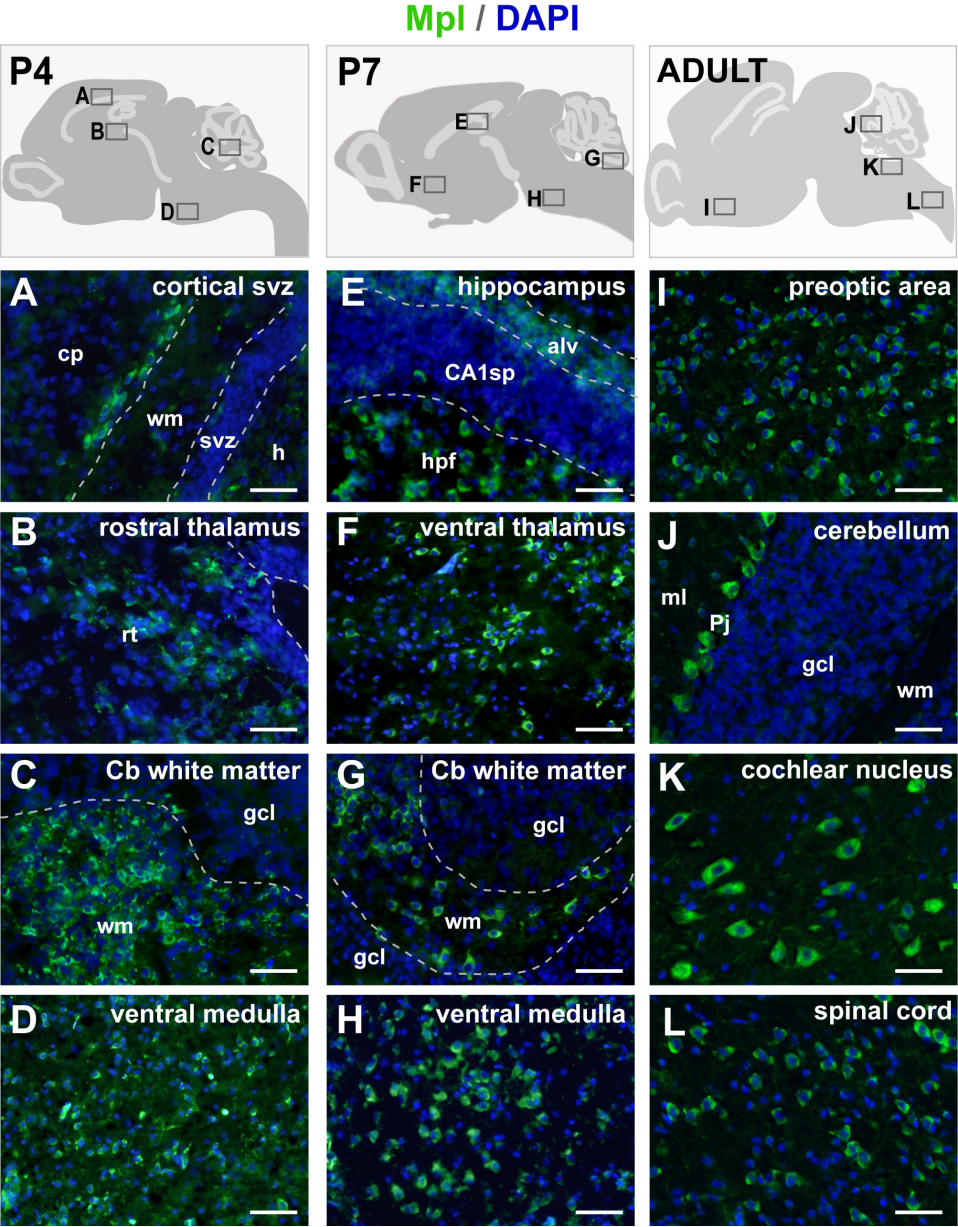
**Mpl protein in the neonatal and adult central nervous system of mice**. Each column combines a schematic drawing to show positions of photographed areas and four sets of pictures. Panel A-D: postnatal stage day 4 (P4); Panel E-H: postnatal stage day 7 (P7); Panel I-L: adult CNS. Dorsal is up, anterior to the left. alv - alveus; CA1sp - cornu ammonis 1 pyramidal layer; cp - cortical plate; hpf - hippocampal formation; gcl - granule cell layer; h - hippocampus; ml - molecular layer; Pj - Purkinje cells; rt - rostral thalamus; svz - subventriclar zone; wm - white matter; scale bar: 50 μm.

At P4, the abundant labelling of the cortical ventricular zone disappeared, leaving behind a few Mpl-positive cells in the interface between the developing white matter and the inner cortical plate (Figure 3A). The subventricular zone surrounding the IV^th ^ventricle had also become Mpl-negative. The expression of all other areas remained almost identical compared to P0 (Figures 3B-D, Table 1)

Three days later, at P7, the number of the Mpl-positive cells further diminished. Interestingly, we observed diffuse labelling in the alveus and in some cells within the hippocampal formation scattered underneath pyramidal cells of CA1 (Figure 3E). Other positive areas included the ventral medulla, pons, cerebellar white matter and various regions of the midbrain. Similarly, Mpl-expression is maintained in most areas of the diencephalon, including thalamus and hypothalamus (Figures 3F-H, Table 1).

Finally, immunohistochemical analysis revealed Mpl-expressing cells also in the adult CNS. Mpl expressing regions in the telencephalon included the cerebral cortex, the hippocampus and the mantle zone of the olfactory bulb (*data not shown*). Large areas in diencephalon (preoptic area; Figure 3I), midbrain and medulla oblongata also contained Mpl positive cells at different density (Table 1). In contrast to the earlier stages, Mpl was detectable in Purkinje cells of the cerebellum, but not longer in the white matter (Figure 3J). Mpl-expressing cells in the brainstem were found in dorsal cochlear nucleus (Figure 3K) and in the grey matter of the spinal cord (Figure 3L).

### Characterization of Mpl expressing cells

**Figure 4:**
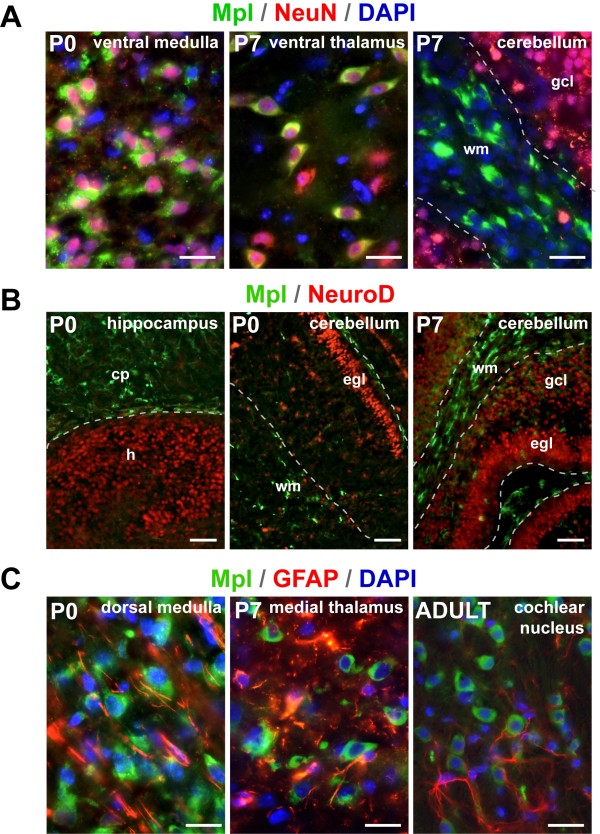
**Characterization of Mpl-positive cells**. (A) Co-localization of Mpl and the pan-neuronal marker NeuN. Almost all Mpl-positive cells (green) are NeuN+ neurons (red) with the exception of cerebellar white matter. (B) Double-labelling of sections for Mpl and NeuroD, an early marker of granule cells. Mpl (green) does not overlap with NeuroD (red) in the cerebellum or in the hippocampus. (C) Expression of Mpl and the astrocyte marker GFAP in different brain regions at P0, P7 and in the adult brain. There is no overlap between Mpl (green) and GFAP (red). cp - cortical plate; egl - external granule layer; gcl - granule cell layer; h - hippocampus; wm - white matter. Scale bars: 20 μm in A and B, 50 μm in C.

To characterize the cells, which express Mpl in the brain, we performed double immunohistochemistry with antibodies directed against Mpl as well as with established markers for neurons and astrocytes. All brain areas on parasagittal sections through the middle of the brain were examined for a possible overlap. The majority of Mpl-positive cells in the developing brain co-expressed the pan-neuronal marker NeuN with one exception: Mpl-expressing cells in the cerebellar white matter did not co-express NeuN (Figure 4A), indicating their possible glial origin. Since the Mpl-positive cells appeared in the brain during the generative period of small granule cells, we also performed double labelling of Mpl with the early granule cell marker NeuroD, but found no overlap in any of the examined regions (see Figure 4B for representative examples).

Moreover, we performed double labelling with the Mpl antibody and the astrocyte marker GFAP to investigate Mpl expression in astrocytes under physiological conditions. However, none of the Mpl-positive cells co-expressed GFAP. Figure 4C shows three representative examples at the developmental stages P0 and P7, as well as in adult mice. Thus, the Mpl-positive cell population in the perinatal and early postnatal murine CNS is mostly neuronal, but definitely heterogenic. For example, Mpl-positive cells in the developing cerebellar white matter had elongated cell bodies and projections parallel to the cerebellar cortex. These cells were negative for both NeuN and GFAP, ruling out the populations of outside-projecting deep cerebellar neurons and astrocytes (Figure 4A). Almost all cells of the developing cerebellar cortex, including granule neurons and small inhibitory neurons were Mpl-negative during perinatal and early postnatal development (Figure 3C and 3G; Figure 4A and 4B). However, in the adult cerebellum, Mpl expression was located in Purkinje cells (Figure 3J).

### Developmental changes in *Mpl *and *Thpo *mRNA expression throughout development

To further clarify the data on the developmental pattern of Thpo/Mpl expression, we analysed expression of both genes in the brain of wild-type mice (Additional file 1). RT-PCR analysis indicated a developmental up-regulation of *Thpo *mRNA. *Mpl *transcript levels appeared to be higher at very early stages (E9.5 to E13.5) compared to the perinatal period and adulthood. However, it needs to be considered that the tissue specimens of early embryonic mice (prior to e15.5) could not be efficiently perfused through the heart for technical reasons. Thus, *Mpl *mRNA levels might be false high, if mRNA from circulating hematopoietic *Mpl *expressing progenitors has been amplified in PCR analysis. This has been experimentally proven by comparing *Mpl *mRNA levels in perfused *vs*. non-perfused specimens of the adult mouse brain (Additional File 1). Beyond E 13.5, however, *Mpl *mRNA levels remained stable, which is concordant with the data obtained by immunohistochemistry.

### Mpl and Thpo mRNA expression in primary neurons, astrocytes, microglia, and neuronal stem cells

**Figure 5:**
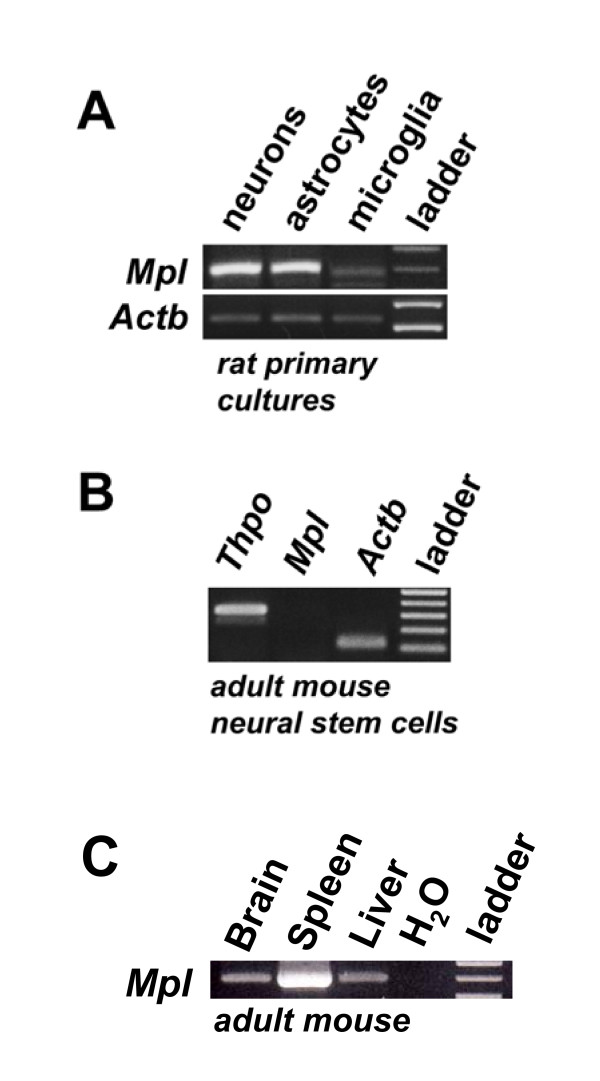
**Expression of *Mpl *mRNA in various primary brain-derived cells**. A. Expression of *Mpl *mRNA as analysed by RT-PCR in neurons, astrocytes and microglia isolated from E17 rat brain. B. Expression of *Thpo*, *Mpl *and *Actb *mRNA in neuronal stem cells of adult mice. C. Expression of *Mpl *transcripts in the brain, spleen and liver of adult mice as controls.

Furthermore, we examined the expression of *Mpl *mRNA in cultured primary brain cells, including neurons, astrocytes, and microglia prepared from the developing rat brain, and in neuronal stem cells prepared from adult mouse brain. Amplification of *Mpl *transcripts showed the presence of *Mpl *expression not only in neurons, but also in cultured astrocytes (Figure 5A). A faint band for *Mpl *transcripts has been found in microglia, most likely resulting from contamination of this culture with astrocytes, which is a normal issue in preparing these cultures. In murine neuronal stem cells, *Thpo *mRNA was highly expressed, while these cells did not express detectable amounts of *Mpl *mRNA (Figure 5B).

### Gross morphology of the Mpl^-/- ^brain

**Figure 6:**
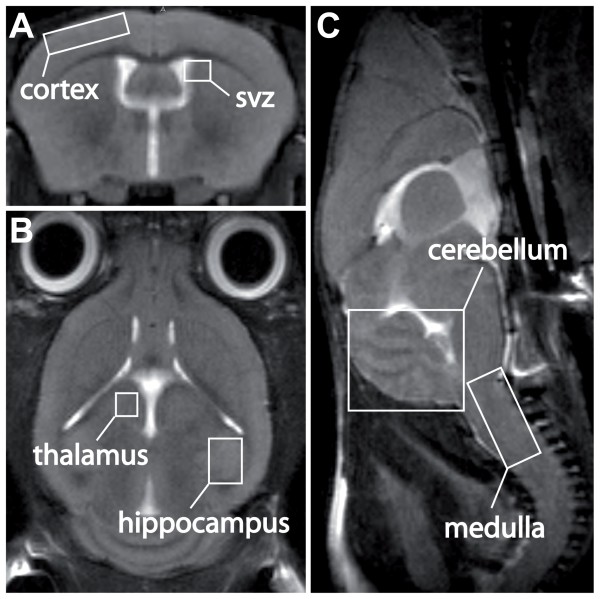
***In vivo *MRI investigation of parenchymal brain abnormalities in *Mpl***^**-/- **^**mice**. Four *Mpl*^-/- ^mice were analysed *in vivo* in two developmental stages (neonatal (P7) and adult) using a standardised high resolution MRI protocol comprising triplanar T2- and T2*-weighted MRI. The cortex as well as the periventricular area including the subventricular zone (SVZ) can be most adequately appreciated in axial slice directions (panel A); the thalami and the hippocampi in coronal slice directions (panel B); the cerebellum and the medulla oblongata in sagittal slice directions (panel C). The brain parenchyma of neither neonatal nor adult *Mpl*^-/- ^mice presented with any gross abnormalities concerning morphology, volume or signal alterations compared to healthy wild type mice.

Investigating the intracranial morphology of *Mpl*^-/- ^mice *in vivo *in neonatal (P7) as well as in adult stages, we did not detect any structural abnormalities on the macroscopic level in comparison to wild-type littermates. All animals presented with normal development and distribution of the white and the grey matter, inconspicuous gyrification, proper width of the inner and outer cerebral fluid interspaces, and normal anatomical morphology of the cerebrum as well as the cerebellum, as presented exemplarily in Figure 6.

## Discussion

To further elucidate the role of the Thpo/Mpl system in the brain, we analysed herein the expression pattern of the Mpl protein in the murine brain by immunohistochemistry. It becomes evident that the Mpl is developmentally regulated, since Mpl is not detectable on protein levels at the very early stages (E12 and E15) of brain development (Figure 2A-C). Mpl protein expression seems to peak around birth (Figure 2D-K) , but is maintained in the adult murine brain (Figure 3; Table 1). Previous data obtained by conventional RT-PCR showed slightly higher *Mpl *mRNA expression in the fetal rat brain compared to adult rat hippocampus and cortex [3]. Considerably high Mpl transcript levels may result in part from circulating hematopoietic cells as shown in our analysis of non-perfused brain tissue specimens (Additional File 1). However, our data also indicate a spatial and temporal expression pattern of Mpl within various areas of the developing and adult brain (Table 1). This may be important for future dissection of the regulation and function of the Thpo/Mpl system in the brain.

During late gestation, Mpl-positive cells are located in the inner layer of the cortex, in the subventricular zone of the IV^th ^ventricle (Figure 2E) and in the olfactory bulb, but not in the hippocampal formation of the telencephalon. Furthermore, Mpl-positive cells are located in various areas of the diencephalon (including thalamus and hypothalamus), in the inferior and superior colliculus of the mesencephalon, in the pons and medulla, and in the grey, but not in the white matter of the spinal cord (Figures 2F-G; Table 1). The lack of Mpl-positive cells in the secondary rhombic lip, which generates the external granule layer of the cerebellum, and in granule cells of the vestibulo-cochlear anlage may indicate that the Thpo/Mpl system is active in cells generated from the residential ventricular zone of the IV^th ^ventricle rather than from the secondary subventricular zone. This hypothesis is supported by the observation that the lateral recesses of the IV^th ^ventricle and granule cell streams, invading into the external cerebellar granule layer and between the cochlear nuclei, remain Mpl-negative at later stages.

During the neonatal period and in adulthood of mice, Mpl expression remains robust in the diencephalon, mesencephalon, myelencephalon and the grey matter of the spinal cord (Table 1). Notably, the strongest variations of Mpl expression occur in the telencephalon and in the metencephalon: In the telencephalon, Mpl-positive cells are initially located in the cortical subventricular zone and in the caudal cortex (Figures 2H, I), but not in the developing white matter. Later, the abundant labelling of cells in the cortical subventricular zone disappears. Only a few Mpl-positive cells can be detected in the interface between the white matter and inner cortical plate at P4 (Figure 3A). However, from P4 onwards, Mpl is expressed in the hippocampus. Mpl receptor is not expressed throughout the hippocampus, but in some multipolar cells in the stratum lacunosum/moleculare. This may be interesting, since the Thpo/Mpl system plays a role in selecting neurons by neurotrophins during ongoing neurogenesis [3].

The second major developmental change in Mpl expression affects the cerebellum. Here, we observe Mpl expression in the cerebellar white matter only during the perinatal period, but no longer in the adult (Figure 3C, G, J; Table 1). Furthermore, Mpl expression in Purkinje cells is obviously silenced during development, but active in the adult cerebellum (Figure 3J). Both observations are of particular interest, since some patients with CAMT or TAR-syndrome, both resulting in impaired Mpl function, exhibit structural and functional abnormalities of the brain, affecting particularly the cerebellum [16,19-21].

Double-labelling with various cell lineage markers suggest that most Mpl expressing cells are neurons, confirmed by staining with the pan-neuronal marker NeuN (Figure 4A). This is consistent with previous data indicating Mpl expression in primary rat hippocampal neurons as well as human SH-SY5Y and rat PC12 cells, which both exhibit a neuronal phenotype [3,8,23]. We could not detect Mpl protein in astrocytes by GFAP, although Mpl has been previously identified in isolated cortical astrocytes by RT-PCR and immunohistochemistry [3]. Thus, we re-evaluated *Mpl *expression in isolated rat astrocytes and could indeed confirm expression of *Mpl *transcripts (Figure 5). Either the sensitivity of immunohistochemistry is too low to detect Mpl expression in brain tissue specimens, or *in vitro *conditions cause up-regulation of *Mpl *mRNA expression. The later hypothesis appears to be more likely, since *Mpl *mRNA expression in astrocytes is known to be induced by hypoxia [3]. The hypoxic up-regulation of Mpl expression in astrocytes contrasts the down-regulation of the Mpl in neuronal cells under the same conditions [3], raising the question whether the Thpo/Mpl system exhibits a different role in astrocytes *vs*. neurons.

Ehrenreich *et al*. identified a proapoptotic role of Thpo that seems to be restricted predominately to immature neuronal cells, suggesting a role for Thpo in the selection of differentiated neurons [3]. In line with this report, we detected *Mpl *mRNA expression in primary cortical neurons, but not in neuronal stem cells (Figure 5). Thus, the combined data support the current model that Thpo exhibits its proapoptotic role in maturating neurons, whereas neuronal stem or progenitor cells are largely unaffected. Thereby, Thpo may indeed help to select neurons that acquire target-derived neurotrophic support [3]. Notably, Mpl up-regulation coincides with a period of very active neurogenesis and establishment of connectivity.

Mpl expression peaks in some crucial brain areas at a period, when normal neurogenesis prunes. This raises the question whether it may render neonates more susceptible than adults to the pro-apopotic effect of Thpo. Such a condition may be of particular significance in the case of intracranial or intraventricular haemorrhage, since it leads to a massive release of circulating Thpo into the brain. Experiments in a mouse model of uni-lateral stroke showed that recombinant Thpo exhibited significantly higher damage scores and extent of apoptosis [3]. To the best of our knowledge, further data on the function of Thpo in any model of ischemic or traumatic brain injury are not available yet.

The biological relevance of the Thpo/Mpl system still needs to be further proven. Due to the developmental changes in Mpl expression in the SVZ of the telencephalon and in the cerebellum (downregulation in the white matter; upregulation in Purkinje cells) as well as structural and functional abnormalities in the brain of some patients with impaired Mpl function (CAMT, TAR syndrome) affecting particularly the cerebellum [16,19-21], we expected structural abnormalities in the brain of mutant mice with homozygous *Mpl *deficiency. Surprisingly, MRI scans of *Mpl*^-/- ^mice did not show gross abnormalities in areas which predominately express the Mpl during brain development or in adulthood. However, ultrasound and/or MRI examinations and assessment of neurodevelopment (for example Bayley Scales of Infant Development) should be undertaken in patients with CAMT or TAR syndrome to get further insights into the role of Thpo and Mpl in the brain. We did not observe a reduced life span of *Mpl*^-/- ^mice. Preliminary data on H&E sections do not indicate cytomorphological changes in the brain of the *Mpl*^-/- ^mice (not shown).

## Conclusions

In summary, our immunohistochemical analysis shows specific expression pattern of Mpl in various regions of the brain. Most strikingly, Mpl expression showed two major developmental changes in the telencephalon (affecting the subventricular zone and the hippocampus) and the cerebellum (affecting the white matter and Purkinje cells). Furthermore, we observed that Mpl protein expression in the brain is largely restricted to neuronal cells and in the adult brain to Purkinje cells of the cerebellum. We could not detect Mpl expression in resting astrocytes, pointing either to expression levels below the detection limit of immunohistochemistry or to an expression limited to certain *in vitro *conditions. The lack of *Mpl *expression in neuronal stem cells and the pattern of Mpl expression in the brain parenchyma support the concept that Thpo is critical for the selection of neurons to undergo apoptosis during a crucial period of neurogenesis. One should be aware of these findings prior to clinical trials on the use of the second generation of thrombopoiesis-stimulating agents in neonates, since these thrombopoietin mimetic peptides or non-peptide molecules are very small in size and molecular weight [24]. Thus, these promising drugs need to be tested whether they cross the blood-brain-barrier in neonates.

## Methods

### Animal experiments

Wild-type CD1 mice (Charles River Laboratories, Sulzfeld) were mated, and noon on the day of discovery of the vaginal plug was designated embryonic day 0 (E0). Mpl expression was analysed on embryonic day 12 (E12), E15, E18, at birth (P0), on postnatal day P1, P4 and P7, as well as in adult mice. Embryos E12-15 were fixed in ice-cold 4% PFA overnight. For better penetration of the fixative, more mature embryos and pups were additionally perfused through the heart under lethal intraperitoneal thiopental anaesthesia. At least 3 animals have been used for each time point. For double-labelling experiments we have examined at least 5 animals per one pair of markers.

Transgenic mice with homozygous deletion of the *Mpl *gene (kindly provided by Warren S. Alexander, Walter & Eliza Hall Institute of medical Research, Melbourne, Australia) were used to validate immunohistochemistry and *in situ *hybridization [13]. The tissue was treated identical to that of wild-type animals. Two animals of each genotype have been examined at three time points. Homozygous *Mpl *deletion was confirmed by PCR analysis of genomic DNA. For PCR analysis, we applied the following primers directed against DNA fragments of the transgenic and the wild-type alleles (GenBank Accession No. NM_001122949): a) 2l Oligo lvg17 5'-TCCAAGGTAAA GCACTGAAGTCCA-3', b) 2l Oligo lvg15 5'-GTCTCCATGGAGGCTTAGGTGGGA-3', and c) 1l Oligo lvg19 5'-GAAGAGCTTGGCGGCGAATGGGCT-3' [13].

All animal procedures fully complied with institutional and state guidelines and were approved by the Institutional Review Board.

### Immunohistochemistry

After overnight cryoprotection of the brain tissue in 20% ice-cold sucrose, the specimens were frozen in OCT (Cryo-M-Bed, Bright Instrument Co. Ltd., Huntingdon, Cambridgeshire, UK) and sectioned at 14 μm. Whole embryos or brains of older animals were cut in parasagittal direction, making twelve series of sections. Two of them containing all sections between the midline and lateral edge of the large hemispheres per animal have been subjected to immunohistochemistry with and without primary antibody and examined under the fluorescent microscope. The sections were washed in PBS containing 0.1% (v/v) Triton X-100 (Sigma), then blocked in PBS containing 0.1% bovine serum albumin Fraction V (Roth, Karlsruhe, Germany), 10% FBS, 0.1% Triton X-100 and 0.05% sodium azide (Sigma). Sections from adult brain have been additionally postfixed with ice-cold 4% PFA/PBS for 10 min and washed three times with PBS containing Triton prior to the permeabilization/blocking step. For immunohistochemical analysis the sections were incubated at 4°C with the following primary antibodies diluted in blocking solution: mouse anti-human MPL (clone 167639; IgG_2a_; R&D Systems Minneapolis, MN; final concentration 2.5 μg/ml), rat anti-mouse Mpl (clone AMM2, 1:200; Kirin Brewery, Tokyo, Japan; final concentration 5 μg/ml), mouse anti-mouse NeuN (A60, IgG_1_; Millipore, Billerica, MA; final concentration 2 μg/ml), goat anti-mouse NeuroD (N-19; Santa-Cruz Biotechnology, Santa Cruz, CA; final concentration 0.4 μg/ml) and rabbit anti-cow GFAP (6F2; DAKO, Glostrup, Denmark; final concentration 5.8 μg/ml). After 3 washes, tissue specimens were incubated with the appropriate secondary antibody for 1 hour at room temperature and counterstained with DAPI (2 μg/ml; Roche, Basel, Switzerland). Cy-2 anti-mouse IgG, Cy-3 anti-goat or anti-rabbit IgG (Jackson ImmunoResearch, West Growe, PA) served as secondary antibodies. The two subclasses of IgGs were differentiated by either Alexa 594 anti-mouse IgG_2a _or Alexa 488 anti-mouse IgG_1 _(Invitrogen,) as secondary antibodies. After detection of the Mpl, sections were thoroughly washed 3 times, fixed with 4% PFA for 5 min to crosslink the signal and processed for the second antibody the same way, including appropriate blocking.

Microscopic analysis was performed using an epifluorescence microscope (AxioPlan 2 Imaging System; Carl Zeiss, Jena, Germany). Photographs were taken with a digital camera (AxioCAM MRc) using AxioVision 4.2 software (Carl Zeiss). While photographing the same region of the knockout animal and its wild-type littermate the same exposure and post processing has been used to allow faithful comparison. One may note that this resulted in a slightly different look of pictures due to deviations in DAPI intensity, but we refrained from adjusting it in Figure 1A.

### *In situ *hybridization

To validate the Mpl antibody, freshly-cut 14 μm cryosections from wild-type and *Mpl*^-/- ^mouse brain were first subjected to *in situ *hybridization with an antisense riboprobe against mouse *Mpl *followed by immunostaining with the anti-human Mpl antibody. *In situ *hybridization was performed as described [25]. The detailed protocol is available at http://www.ucl.ac.uk/~ucbzwdr/richardson.htm. The digoxigenin-labeled riboprobe was transcribed from a ~2kb cDNA encoding the full-length murine *Mpl *cDNA (GenBank Accession No. NM_001122949) cloned into pSPT18 vector (kindly provided by Michelle Souyri, Hôpital Paul Brousse Villejuif, France) [26].

### Preparation of primary cells from the brain

Primary neuronal cultures of cerebral cortex from Wistar rat embryos (E17) were prepared as previously described [27]. Astroglial and microglial cell cultures were prepared from newborn rats as described elsewhere [28]. Microglial cells were seeded at a density of 100,000 cells/cm^2 ^and harvested after 24 h. Astrocytes were seeded at a density of 120,000 cells/cm^2 ^and harvested after 48 h. Adult neural stem cells (NSC) were prepared from C57/Bl6 mice as described [29]. NSC were cultivated in NBMA (Invitrogen) containing 2% B27 without retinoic acid (Invitrogen), 1% L-glutamine, 10 ng/ml bFGF, and 20 ng/ml EGF (Biochrom, Berlin, Germany).

### RT-PCR analysis of Mpl expression

*Mpl *mRNA expression was analysed in primary brain-derived cells by conventional RT-PCR analysis. Total RNA was prepared using TRIzol reagent^® ^(Invitrogen), and first strand cDNA synthesis was performed with 2 μg of total RNA using oligo(dt) primers (Promega,) and M-MLV reverse transcriptase (Invitrogen). One-twentieth of reaction product was used for PCR amplification in a thermal cycler, which was carried out under the following conditions: DNA denaturation at 94°C for 60 seconds, primer annealing at 60°C (or 55°C rat Mpl) for 60 seconds, extension of double-stranded DNA at 72°C for 90 seconds. The following primers were used: mouse or rat *Mpl *5'-CTAGCTCCCAAGGCTTCTTC-3' (forward primer), 5'-GGCTCCAGCACCTTCCAGTCC -3' (reverse primer), product size 392 bp (GenBank Accession No. NM_001122949); mouse *Thpo *5'-CTCTGTCCAGCCCCGTAGC-3' (forward primer), 5'-CCCCAA GAGGAGGCGAAC-3' (reverse primer), product size 314 bp (GenBank Accession No. NM_009379); mouse *Actb *5'-ACTGCTCTGGCTCCTAGCAC-3' (forward primer), 5'-ACATCTGCTGGA AGGTGGAC-3' (reverse primer), product size 115 bp (GenBank Accession No. NM_007393); rat *Actb *5'-ATCGTGGGCCGCCTAGCACC-3' (forward primer), 5'-CTCTTTAATGTCACGCACGATTTC-3' (reverse primer), product size 542 bp (GenBank Accession No. NM_031144). Amplified PCR fragments were visualized on 1.5% agarose gel stained with ethidium bromide.

### MRI analysis

Cerebral MRI was performed on a 7 Tesla rodent MRI scanner (Pharmascan 70/16AS, Bruker BioSpin, Ettlingen, Germany), applying a 20 mm RF-Quadrature-Volume head coil. Animals received anesthesia via facemask induced with 3% and maintained with 1.5 - 2.0% isoflurane (Forene, Abbot, Wiesbaden, Germany) delivered in 100% O_2 _under constant ventilation control (Bio Trig System, Bruker BioSpin, Ettlingen, Germany). Mice were placed on a heated circulating water blanket to keep up body temperature at 37°C. IRB protocol G0240/06, Landesamt für Gesundheit und Soziales, Berlin, Germany.

Triplanar fat-suppressed turbo spin echo T2-weighted (RARE; TE1 14.5 ms, TE2 65.5 ms, TR 4500 ms, 0.5 mm slice thickness, Matrix 256 × 256, FOV 2.8 cm, eight averages, 40 coronal slices, scan time 28 minutes, and 20 axial slices, scan time 28 minutes) and T2*-weighted (GEFI; TE 5.6 ms, TR 1200 ms, flip angle 35°, 0.5 mm slice thickness, Matrix 256 × 256, FOV 2.8 cm, four averages, 40 coronal slices, scan time 20 minutes, and 20 axial slices, scan time 13 minutes) images were acquired. Identical slice positions were used for all sequences applied: coronal slices were aligned to the olfactory bulb/frontal lobe fissure and covered the entire brain up to the cervical spinal cord. Axial slices were positioned parallel to a plane through the most frontal tip of the olfactory bulb and the most rostral cerebellar part. MRI data were analysed using the MEDx3.4.3 software package (Medical Numerics, Virginia, USA) on a LINUX workstation.

## Authors' contributions

AI established, performed and analyzed *in situ *hybridizations as well as immunohistochemistry; AI also drafted and revised the manuscript. JW performed MRI and edited the manuscript. JZ performed Mpl expression analysis by PCR technique. OH isolated neuronal stem cells and revised the manuscript. MB provided the *Mpl*^-/- ^mice, various experimental tools and revised the manuscript. CD designed the project, supervised the experimental work and wrote the manuscript. All authors read and approved the final version of the manuscript.

## Supplementary Material

Additional file 1 **Analysis of expression of *Thpo-mRNA *and *Mpl-mRNA *during the development of mouse brain**. Conventional RT-PCR analysis of *Mpl *and Thpo mRNA expression in the developing and adult brain. Concerning relatively high *Mpl *transcript levels during early embryonic development, one might take into account that the tissues overlying the developing central nervous system contain circulatory hematopoietic cells that carry Mpl. Transcardiac perfusion, often used to minimize that, cannot be efficiently used before E15 for technical reasons. This is supported by higher *Mpl *transcript levels in non-perfused *vs*. perfused brain specimens. Click here for file
