# Supplementary figures and images for: Expression pattern of the thrombopoietin receptor (Mpl) in the murine central nervous system
Source: BMC Dev Biol. 2010 Jul 28;10:77. doi: 10.1186/1471-213X-10-77 (PMC2921376; doi:10.1186/1471-213X-10-77)

## Supplemental Figure 1.

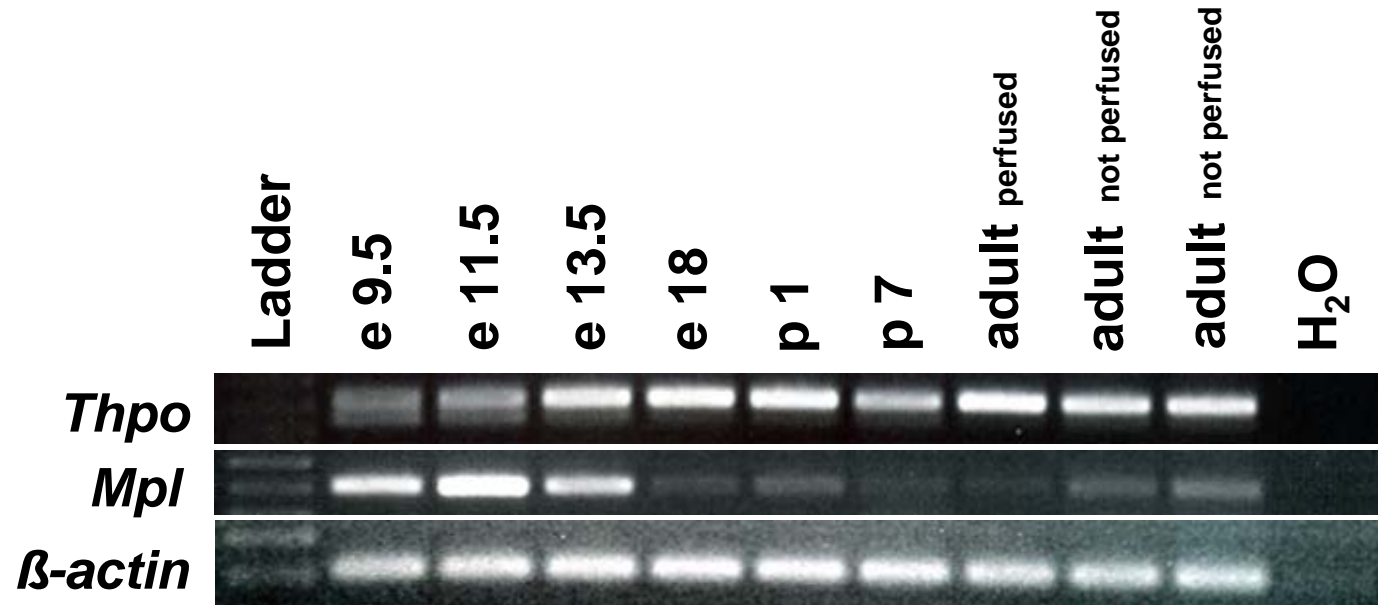

Supplement: Additional file 1 — Analysis of expression of Thpo-mRNA and Mpl-mRNA during the development of mouse brain. Conventional RT-PCR analysis of Mpl and Thpo mRNA expression in the developing and adult brain. Concerning relatively high Mpl transcript levels during early embryonic development, one might take into account that the tissues overlying the developing central nervous system contain circulatory hematopoietic cells that carry Mpl. Transcardiac perfusion, often used to minimize that, cannot be efficiently used before E15 for technical reasons. This is supported by higher Mpl transcript levels in non-perfused vs. perfused brain specimens. [file 1471-213X-10-77-S1.PDF]
